# DCAF1 controls T-cell function via p53-dependent and -independent mechanisms

**DOI:** 10.1038/ncomms10307

**Published:** 2016-01-05

**Authors:** Zengli Guo, Qing Kong, Cui Liu, Song Zhang, Liyun Zou, Feng Yan, Jason K. Whitmire, Yue Xiong, Xian Chen, Yisong Y. Wan

**Affiliations:** 1 Lineberger Comprehensive Cancer Center, University of North Carolina at Chapel Hill, Chapel Hill, North Carolina 27599, USA; 2 Department of Microbiology and Immunology, University of North Carolina at Chapel Hill, Chapel Hill, North Carolina 27599, USA; 3 Deparment of Biochemistry and Biophysics, University of North Carolina at Chapel Hill, Chapel Hill, North Carolina 27599, USA; 4 Department of Genetics, University of North Carolina at Chapel Hill, Chapel Hill, North Carolina, 27599 USA; 5 Jiangsu Center for the Collaboration and Innovation of Cancer Biotherapy, Cancer Institute, Xuzhou Medical College, Xuzhou, Jiangsu 221002, China

## Abstract

On activation, naive T cells grow in size and enter cell cycle to mount immune response. How the fundamental processes of T-cell growth and cell cycle entry are regulated is poorly understood. Here we report that DCAF1 (Ddb1–cullin4-associated-factor 1) is essential for these processes. The deletion of DCAF1 in T cells impairs their peripheral homeostasis. DCAF1 is upregulated on T-cell receptor activation and critical for activation-induced T-cell growth, cell cycle entry and proliferation. In addition, DCAF1 is required for T-cell expansion and function during anti-viral and autoimmune responses *in vivo*. DCAF1 deletion leads to a drastic stabilization of p53 protein, which can be attributed to a requirement of DCAF1 for MDM2-mediated p53 poly-ubiquitination. Importantly, p53 deletion rescues the cell cycle entry defect but not the growth defect of DCAF1-deficient cells. Therefore, DCAF1 is vital for T-cell function through p53-dependent and -independent mechanisms.

## Abstract (web summary)

DCAF1 (VprBP), an HIV target protein, is a component of an ubiquitin ligase complex involved in developmental processes in plants and animals. Here, DCAF1 is shown to be vital for activation-induced T cell growth and proliferation, partly by being required for p53's ubiquitination and degradation.

## Graphical abstract

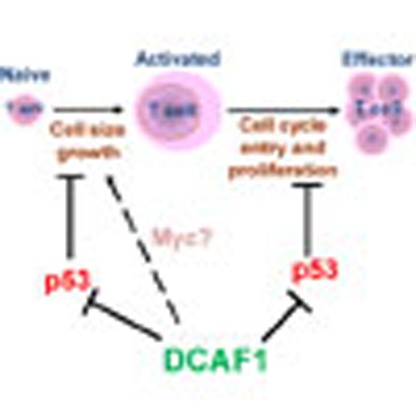

Mature naive T cells in the periphery remain quiescent with virtually undetectable cell growth (biomass increase due to elevated metabolism and biosynthesis) or proliferation under steady state. On antigenic stimulation, naive T cells grow in size followed by cell cycle entry and rapid proliferation to become effector T cells^1^,^2^. When the antigenic stimuli diminish, most of the effector T cells die and the others return to quiescence. The quiescent effector/memory T cells readily enter cell cycle and to proliferate when they are re-stimulated with antigen and mitogen. Activation-induced cell cycle entry and proliferation of T cells are central for T-cell-mediated immune response. Deregulated T-cell proliferation often leads to debilitating and fatal diseases including immune deficiency, chronic inflammation, autoimmunity and leukaemia^3^,^4^. Nonetheless, how T-cell receptor (TCR) signalling and cell cycle regulation are coordinated remains largely unaddressed. Specifically, how the fundamental processes of activation induced T-cell growth and cell cycle entry are regulated is poorly understood. We therefore investigated factors upregulated by TCR activation in T cells and found DCAF1 expression to be highly induced.

DCAF1, also known as VprBP (HIV-1 viral protein r-binding protein), is an evolutionary conserved substrate-binding subunit of CRL4 (Cul4a–Ddb1–Roc1) ubiquitin ligase complex^5^,^6^,^7^,^8^. It is a cellular protein targeted by HIV-1 viral protein R (Vpr)^9^. The function of DCAF1 in mammalian cells attracted much attention recently. DCAF1 modulates cellular response against HIV in macrophages^10^,^11^, controls the survival and reprogramming of oocyte^12^,^13^, and regulates G_2_/M transition^14^,^15^,^16^,^17^,^18^,^19^,^20^,^21^,^22^. DCAF1 functions through diverse mechanisms including regulating protein poly-ubiquitination, mono-ubiquitination and phosphorylation^13^,^23^,^24^,^25^. Nonetheless, whether and how DCAF1 controls the function of primary T cell, a major cell type infected by HIV^26^, remains unknown.

We studied the role for DCAF1 in T cells by deleting DCAF1 specifically in these cells. The disruption of DCAF1 impairs the peripheral maintenance of T cells. DCAF1 is required for the cell growth and cell cycle entry of activated T cells. DCAF1 deletion abrogates T-cell expansion during anti-viral and autoimmune responses *in vivo*. DCAF1-deficient cells drastically stabilize p53 protein with elevated expression of p53 target genes, which is attributed to a requirement of DCAF1 for MDM2-promoted p53 poly-ubiquitination. Intriguingly, we found that although p53 deletion failed to correct the T-cell growth defect due to DCAF1 deletion, it restored the cell cycle entry of DCAF1-deficient T cells. These findings demonstrate an essential role for DCAF1 in T-cell homeostasis and the growth and cell cycle entry of activated T cells. In addition, this study identifies p53 a critical downstream factor of DCAF1 in controlling cell cycle entry.

## Results

### DCAF1 expression in naive and activated T cells

**Figure 1:**
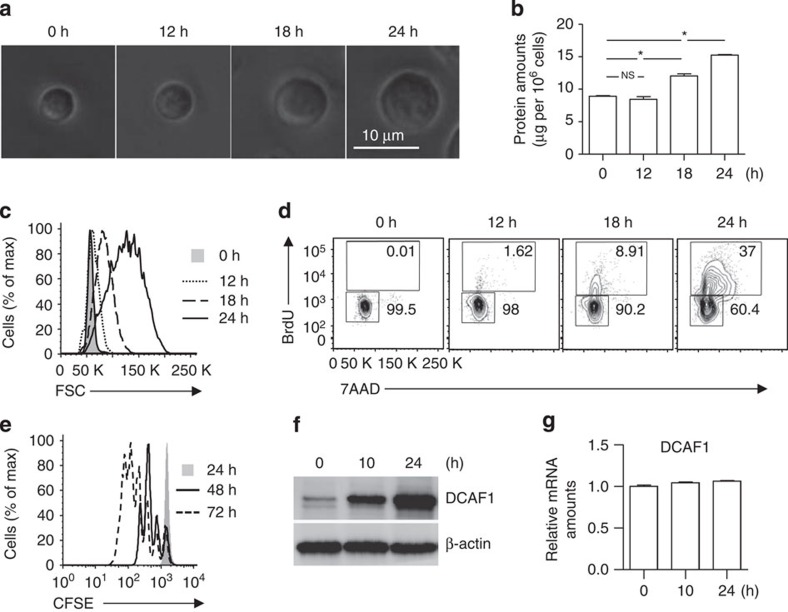
Activation-induced cell growth and cell cycle entry of T cells. (**a**–**c**) Naive CD4^+^ T cells were activated by anti-CD3 and anti-CD28. At indicated time points after activation, cell growth was analysed by microscopy for morphology (**a**), by bicinchoninic acid assay (BCA) assay for protein amount (**b**), and by flow-cytometry for cell size (**c**). Means±s.d. of three experiments are shown (*P<0.05 by Student's *t-*test; NS, not significant, *P*>0.05 by Student's *t*-test). Representative results of at least three independent experiments are shown. (**d**,**e**) The amount of DNA synthesis was determined by BrdU incorporation (**d**), and the proliferation was assessed by CFSE dilution (**e**) of CD4^+^ naive T cells activated by anti-CD3 and anti-CD28 at indicated time points. Results are representative of three experiments. (**f**,**g**) The expression of DCAF1 was monitored by immunoblotting (**f**) and quantitative reverse transcription–PCR (**g**) assays at indicated time points after CD4^+^ naive T cells were activated by anti-CD3 and anti-CD28. Results are representative of three experiments.

To identify factors upregulated during T-cell activation, we purified naive CD4 T cells from wild-type mice and stimulated them with anti-CD3 and anti-CD28. We found that, on TCR ligation, T-cell size grew gradually to reach a twofold increase in diameter by 24 h (Fig. 1a), associating with elevated protein contents (Fig. 1b). The cell growth could be readily detected by flow-cytometry (Fig. 1c). The hallmark for cell cycle entry is the DNA synthesis and subsequent cell division. Using BrdU incorporation assay, we found that while the DNA synthesis of non-activated naive T cells was virtually undetectable that of TCR-activated T cells started at 12 h, became evident at 18 h and reached a high level at 24 h post activation (Fig. 1d), followed by rapid cell division (Fig. 1e). Using this experimental system, we identified factors upregulated by TCR stimulation and found that the protein expression of DCAF1 was low in naive T cells and drastically upregulated during activation-induced T-cell growth and cell cycle entry (Fig. 1f), although the mRNA expression appeared largely unchanged (Fig. 1g). We therefore hypothesized that DCAF1 was involved in activated T-cell function.

### The development and homeostasis of *Cd4Cre*;*Dcaf1* ^fl/fl^ T cells

**Figure 2:**
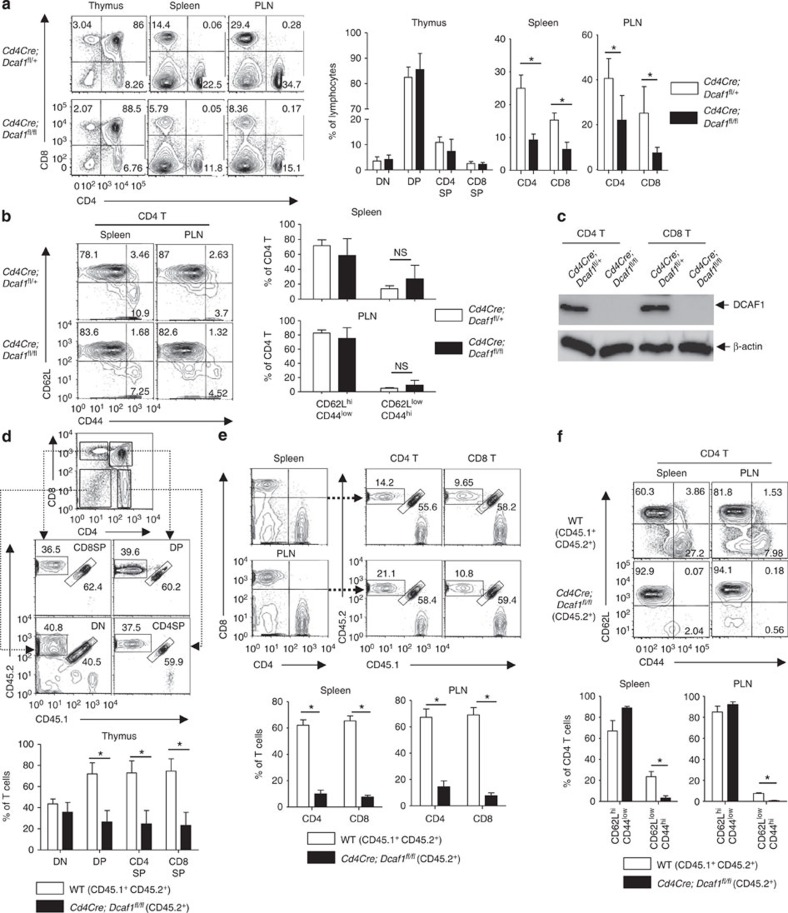
DCAF1 regulates T-cell development and homeostasis. (**a**) The distribution of various T-cell populations in the thymus, spleen and periphery lymph nodes (PLN) of mice with different genotypes, assessed by flow-cytometry. The bar graphs show the means±s.d. of four sets of mice (*P<0.05 by Student's *t-*test). (**b**) The distribution of naive (CD62L^high^CD44^low^) and effector/memory (CD62L^low^CD44^high^) CD4^+^ T cells in the spleen and periphery lymph node (PLN) of mice with different genotypes, assessed by flow-cytometry. The bar graphs show the means±s.d. of four sets of mice (NS, not significant, *P*>0.05 by Student's *t-*test). (**c**) DCAF1 protein expression in CD4^+^ and CD8^+^ T cells sorted from mice of different genotypes, analysed by immunoblotting. The immunoblotting is representative of at least three experiments. (**d**) The comparison of the thymocyte populations in the irradiated recipient chimeric mice (CD45.1^+^) reconstituted with equal numbers of bone marrow cells from wild-type mice (CD45.1^+^CD45.2^+^) and *Cd4Cre;Dcaf1*^fl/fl^ mice (CD45.2^+^). The bar graphs show the means±s.d. of data from five recipient mice (*P<0.05 by Student's *t-*test). (**e**) The comparison of CD4^+^ and CD8^+^ T populations in the spleens and periphery lymph nodes (PLN) of the recipient chimeric mice. The bar graphs show the means±s.d. of data from five recipient mice (*P<0.05 by Student's *t-*test). (**f**) The comparison of naive (CD62L^high^CD44^low^) and effector/memory (CD62L^low^CD44^high^) CD4^+^ T cells in the spleens and periphery lymph nodes (PLN) of the recipient chimeric mice. The bar graphs show the means±s.d. of data from five recipient mice (*P<0.05 by Student's *t-*test). See also Supplementary Fig. 1.

To study the function of DCAF1 in T cells, we deleted DCAF1 specifically in mature T cells by crossing *Cd4Cre* mice^27^ with *Dcaf1*^fl/fl^ mice^15^. *Cd4Cre*;*Dcaf1*^fl/fl^ mice were born at the Mendelian ratio and phenotypically grossly normal. T-cell development was largely normal in *Cd4Cre*;*Dcaf1*^fl/fl^ mice. Compared with *Cd4Cre*;*Dcaf1*^fl/+^ littermates, *Cd4Cre*;*Dcaf1*^fl/fl^ mice had similar percentages of double negative (DN) and double positive, but slightly less CD4SP (single positive) and CD8SP thymocytes (Fig. 2a) that express similar amounts of maturation markers (Supplementary Fig. 1a–c). The percentages and numbers of mature CD4^+^ and CD8^+^ T cells in the peripheral lymphoid organs were lower in *Cd4Cre*;*Dcaf1*^fl/fl^ than in *Cd4Cre*;*Dcaf1*^fl/+^ mice (Fig. 2a and Supplementary Fig. 1d). Nonetheless, abundant numbers of naive (CD62L^high^CD44^low^) mature T cells could be recovered from *Cd4Cre*;*Dcaf1*^fl/fl^ mice (Fig. 2b, Supplementary Fig. 1d,e) with efficient deletion of DCAF1 protein (Fig. 2c). These findings suggest DCAF1 deletion affects the homeostasis of mature T cells.

To further investigate whether DCAF1 is required for T-cell peripheral maintenance via a cell-intrinsic mechanism, we generated mixed-bone-marrow chimeric mice where wild-type (CD45.1^+^CD45.2^+^) and *Cd4Cre*;*Dcaf1*^fl/fl^ (CD45.2^+^) T cells were generated and maintained in the same host (CD45.1^+^). We found that *Cd4Cre*;*Dcaf1*^fl/fl^ thymocyte development was moderately defective in the chimera mice (Fig. 2d) and that DCAF1-deficient mature T cells were poorly maintained in the periphery when compared with wild-type cells (Fig. 2e). We also noticed that, compared with co-existing wild-type T cells, DCAF1-deficient mature T cells in the chimeras were composed of predominantly naive (CD62^high^CD44^low^) and much fewer effector/memory (CD62L^low^CD44^high^) T cells (Fig. 2f and Supplementary Fig. 1f), suggesting that DCAF1 deletion was more detrimental to the maintenance of effector/memory T cells than that of naive T cells. The relatively normal distribution of effector/memory T-cell population in *Cd4Cre*;*Dcaf1*^fl/fl^ mice (Fig. 2b) was therefore likely due to the lymphopenia-drive aberrant T-cell activation^28^. These findings thus demonstrate that DCAF1 is required for T-cell homeostasis in a cell-intrinsic manner.

### DCAF1 is vital for activated T cell growth and cell cycle entry

**Figure 3:**
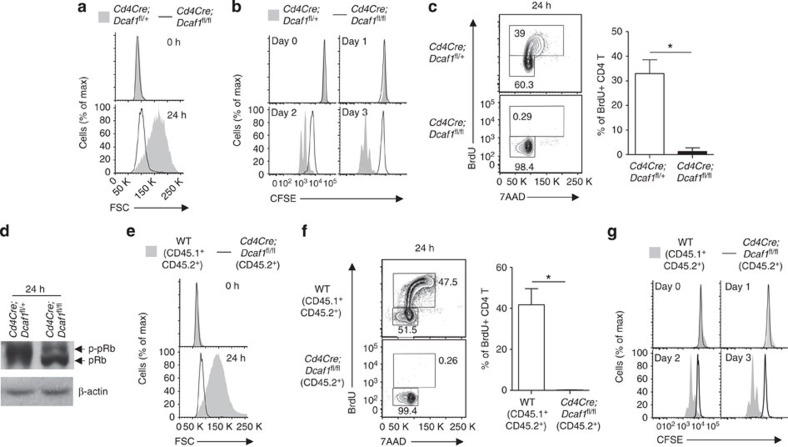
DCAF1 is required for cell growth and cell cycle entry. (**a**–**d**) The comparison of the sizes (**a**), the proliferation (**b**), the DNA synthesis (**c**) and the amount of Rb protein (pRb) and its phosphorylation (p-pRb) (**d**) of CD4^+^ naive T cells with different genotypes at indicated time points after being activated with anti-CD3 and anti-CD28. The bar graphs show the means±s.d. of data from five experiments (*P<0.05 by Student's *t-*test). (**e**–**g**) The comparison of the cell sizes (**e**), the DNA synthesis (**f**) and the proliferation (**g**) of CD4^+^ T cells of different genotypes co-existing in the mixed-bone-marrow chimeric mice described in Fig. 2d–f at indicated time points after TCR activation. The bar graphs show the means±s.d. of data from four experiments (*P<0.05 by Student's *t-*test). See also Supplementary Fig. 2.

The upregulation of DCAF1 on TCR stimulation (Fig. 1f) indicated its potential importance for activation-induced T-cell growth and cell cycle entry. To test this, we purified naive CD4 T cells from wild-type and *Cd4Cre*;*Dcaf1*^fl/fl^ mice and activated them with anti-CD3 and anti-CD28. We found that DCAF1-deficient naive T cells failed to enlarge in size (Fig. 3a, Supplementary Fig. 2a) or to increase cellular protein amounts after TCR activation (Supplementary Fig. 2b), although the upregulation of T-cell activation markers, including CD69, CD25 and CD44, was comparable between wild-type and DCAF1-deficient T cells (Supplementary Fig. 2c). In addition, the proliferation of DCAF1-deficient T cells was completely abolished (Fig. 3b). While DCAF1 has been shown to be involved in G_2_/M transition during cell cycle^14^,^15^,^16^,^17^,^18^,^19^,^20^,^21^,^22^, the proliferation defect observed in DCAF1-deficient T cells appeared primarily due to defective cell cycle entry. In the absence of DCAF1, the S-phase entry did not occur in activated naive T cells (Fig. 3c), associating with reduced cyclin expression (Supplementary Fig. 2d) and Rb hyper-phosphorylation (Fig. 3d), a critical step required for cell cycle entry^29^,^30^. Besides abolished T-cell growth and cell cycle entry, activated DCAF1-deficient T cells were more prone to apoptosis than wild-type cells (Supplementary Fig. 2e).

The decreased T-cell number in *Cd4Cre*;*Dcaf1*^fl/fl^ mice (Fig. 2a) may lead to a partial lymphopenic environment that could adversely affect T-cell function^28^. To study the cell-intrinsic defect of DCAF1-deficient T cells, we compared the functions of wild-type and DCAF1-deficient T cells developed together in the mixed-bone-marrow chimera (described in Fig. 2d–f). Compared with co-existing wild-type T cells, DCAF1-deficient T cells failed to grow in size after TCR activation (Fig. 3e), although they upregulated T-cell activation markers normally (data not shown). In addition, the proliferation and the cell cycle entry of DCAF1-deficient T cells were abolished (Fig. 3f,g). These findings demonstrated that DCAF1 is intrinsically required for T-cell growth and cell cycle entry in response to TCR activation.

### Cytokine-stimulated cell cycle entry requires DCAF1

Cell growth occurs slightly earlier than cell cycle entry in naive T cells after TCR activation (Fig. 1a–d). Thus, the defect of cell cycle entry observed in DCAF1-deficient naive T cells (Fig. 3c,f) could be a consequence of failed T-cell growth (Fig. 3a,e). To address whether DCAF1 is specifically required for cell cycle entry of activated T cells, we used effector T-cell re-stimulation model. In this model, naive T cells were activated by anti-CD3 and anti-CD28 under culturing conditions. Five days later, most of the activated T cells ceased cell cycle and returned to quiescent state with minimal DNA synthesis when no further stimulation was provided (Supplementary Fig. 3a). On cytokine IL-2 re-stimulation, the quiescent effector T cells re-entered cell cycle with a high rate of DNA synthesis (Supplementary Fig. 3a) but without obvious cell growth (Supplementary Fig. 3b,c). Therefore, re-stimulation of quiescent effector T cells can be used as a model to specifically study the cell cycle entry independent of cell growth.

**Figure 4:**
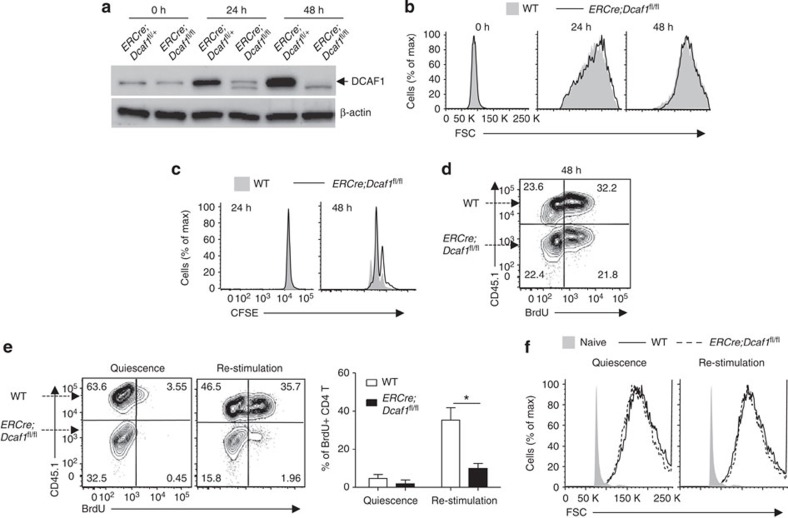
DCAF1 is essential for cell cycle entry. (**a**) DCAF1 protein expression in CD4^+^ T cells of different genotypes at indicated time points after TCR activation and 4-hydroxy-tamoxifen treatment, analysed by immunoblotting. The immunoblotting is representative of at least three experiments. (**b**–**d**) Equal numbers of wild-type (CD45.1^+^) and *ERCre*;*Dcaf1*^fl/fl^ (CD45.2^+^) CD4^+^ T cells were mixed and activated with anti-CD3 and anti-CD28 in the presence of 4-hydroxy-tamoxifen. At indicated time points after activation, the cell sizes (**b**), the proliferation (**c**) and the amount of DNA synthesis (measured by BrdU incorporation assay) (**d**) of the T cells of different genotypes were assessed and compared. Results are representative of at least three experiments. (**e**,**f**) CD4^+^ T cells from wild-type (CD45.1^+^) and *ERCre*;*Dcaf1*^fl/fl^ (CD45.2^+^) mice were mixed and activated by anti-CD3 and anti-CD28 in the presence of 4-hydroxy-tamoxfin for 5 days for them to become effector T cells. Quiescent effector T cells were either re-stimulated with IL-2 or remained unstimulated (quiescence). The amount of DNA synthesis (measured by BrdU incorporation assay) (**e**) and the sizes (**f**) of the cells of different origins were compared. The bar graphs show the means±s.d. of data from four experiments (*P<0.05 by Student's *t-*test). See also Supplementary Fig. 3.

To investigate if DCAF1 is required for cell cycle entry of effector T cells, we crossed *Dcaf1*^fl/fl^ mice^15^ with tamoxifen-inducible-Cre (*ERCre*) mice^31^ to delete DCAF1 at the time of our choice. On 4-hydroxy-tamoxifen treatment *in vitro*, the protein expression of DCAF1 in *ERCre;Dcaf1*^fl/fl^ T cells was greatly reduced by 24 h and abolished by 48 h after TCR activation (Fig. 4a). We then compared cell growth and proliferation of mixed wild-type (CD45.1^+^) and *ERCre;Dcaf1*^fl/fl^ (CD45.2^+^) T cells following TCR activation under the same culturing conditions. Because cell growth occurred before DCAF1 was fully deleted, the sizes of wild-type and *ERCre;Dcaf1*^fl/fl^ T cells were comparable (Fig. 4b). In addition, the rates of cell division (Fig. 4c) and DNA synthesis (Fig. 4d) were slightly lower in *ERCre;Dcaf1*^fl/fl^ than in wild-type T cells. Therefore, this experimental system allows us to generate DCAF1-deficient effector T cells to study whether DCAF1 is required for IL-2-stimulated cell cycle entry in effector T cells as described in Supplementary Fig. 3. We found that while wild-type (CD45.1^+^) effector T cells readily entered cell cycle on IL-2 re-stimulation, DCAF1-deficient T cells (CD45.2^+^) failed to synthesize DNA under the same conditions (Fig. 4e). Such a requirement of DCAF1 for cell cycle entry of T cells appeared to be independent of cell growth because the sizes of wild-type and DCAF1-deficient T cells were virtually identical during quiescence and after IL-2 re-stimulation (Fig. 4f). These results suggest that DCAF1 controls cell cycle entry.

### DCAF1 controls T cell responses *in vivo*

**Figure 5:**
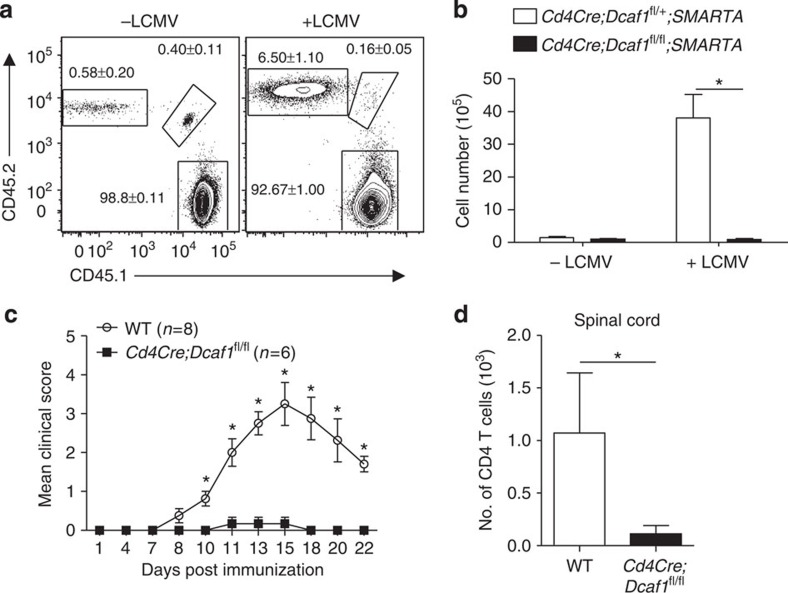
DCAF1 controls T-cell response during viral infection and autoimmunity. (**a**,**b**) 1 × 10^6^ of CD4^+^ T cells isolated from *Cd4Cre;Dcaf1*^fl/+^*;SMARTA* mice (CD45.2^+^) and *Cd4Cre;Dcaf1*^fl/fl^*;SMARTA* mice (CD45.1^+^CD45.2^+^) were mixed and transferred into syngeneic wild-type recipient mice (CD45.1^+^) followed by LCMV infection (+LCMV) or remain uninfected (−LCMV). The percentages (**a**) and the numbers (**b**) of donor cells of different genotypes were determined by flow-cytometry 5 days after infection. Representative results and means±s.d. of 10 mice of 2 experiments are shown (*P<0.05 by Student's *t-*test). (**c**,**d**) Wild-type (WT) (*n*=8) and *Cd4Cre;Dcaf1*^fl/fl^ (*n*=6) mice were injected with MOG/CFA to elicit autoimmune encephalomyelitis (EAE). The disease clinic scores were recorded at indicated time points. Means±s.d. are shown (**c**) (*P<0.05 by Mann–Whitney's *U*-test). The numbers of spinal cord infiltrating CD4^+^ T cells were assessed 22 days post EAE elicitation. Means±s.d. are shown (**d**) (*P<0.05 by Student's *t-*test).

Antigen-induced clonal expansion is critical for T-cell immunity. Aforementioned findings in cell culture condition encouraged us to investigate whether DCAF1 was required for T-cell clonal expansion and function *in vivo* during infection and autoimmunity. We first addressed if DCAF1 deletion affected T-cell clonal expansion in response to lymphocytic choriomeningitis virus (LCMV) infection. We crossed *Cd4Cre*;*Dcaf1*^fl/fl^ mice with SMARTA TCR transgenic mice, whose CD4^+^ T cells can be specifically activated by LCMV peptide GP_61–80_ (ref. 32). CD4^+^ T cells from *Cd4Cre*;*Dcaf1*^fl/+^;*SMARTA* mice (CD45.2^+^) and *Cd4Cre*;*Dcaf1*^fl/fl^;*SMARTA* mice (CD45.1^+^CD45.2^+^) were purified, mixed at the ratio of 1:1, and then transferred into syngeneic wild-type recipient mice (CD45.1^+^). Recipient mice were then infected with LCMV (Amstrong strain) to activate transferred T cells. While DCAF1-sufficient SMARTA T cells expanded dramatically in response to LCMV infection; DCAF1-deficient SMARTA T cells failed to expand in the same hosts (Fig. 5a,b).

We next examined whether DCAF1 controls T-cell function in an autoimmune disease model, experimental autoimmune encephalomyelitis (EAE), by immunizing mice with myelin oligodendrocyte (MOG) peptide emulsified in complete Freund's adjuvant (CFA). While wild-type mice developed EAE, *Cd4Cre*;*Dcaf1*^fl/fl^ mice were refractory to MOG/CFA-induced EAE (Fig. 5c). Further analysis showed that much less T cells were recovered from the spinal cords of *Cd4Cre*;*Dcaf1*^fl/fl^ mice when compared with wild-type mice (Fig. 5d). These results indicate that DCAF1 is essential for T-cell clonal expansion *in vivo* and for T-cell-mediated anti-viral and autoimmune response.

### DCAF1 interacts with COP9 signalosome

**Figure 6:**
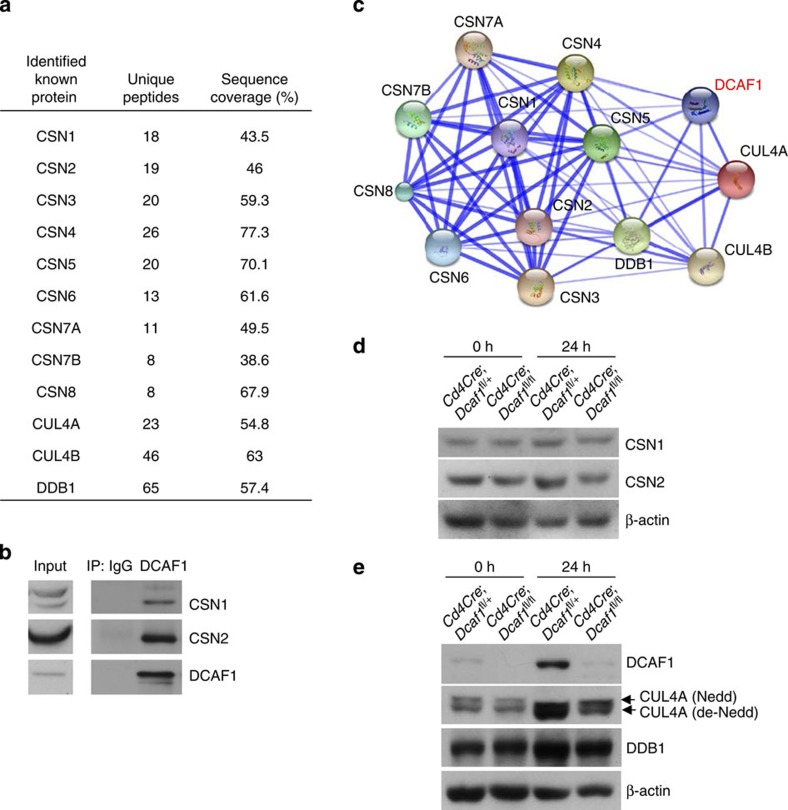
DCAF1 interacts with COP9 signalosome. (**a**) The proteins preferentially immunoprecipitated by anti-DCAF1 over anti-IgG in TCR-stimulated CD4^+^ T cells were identified by mass spectrometry. COP9 signalosome components and CRL4 subunits identified were listed in the table (**a**), including the number of unique peptides (Unique Peptides) detected and the percentage of sequence coverage (Sequence Coverage). Results were obtained from three experiments. (**b**) Co-immunoprecipitation of CSN1, CSN2 with DCAF1 in TCR-stimulated CD4^+^ T cells. (**c**) The protein–protein interaction network of DCAF1 containing CRL4–COP9 mega complex was generated using the STRING database (Version 9.1, http://string-db.org/), based on the proteins identified in **a**. (**d**,**e**) The protein expression of CSN1, CSN2, DDB1, CUL4A and DCAF1 was determined in CD4^+^ T cells of different genotypes before (0 h) and 24 h after TCR stimulation. All results are representative of three independent experiments. See also Supplementary Table 1.

The findings that DCAF1 is required for cell size growth and cell cycle entry from quiescence promoted us to investigate the underlying molecular mechanisms. To identify the cellular factors that associate with DCAF1 in T cells, we analysed DCAF1-interacting proteins using mass spectrometry (MS). DCAF1 co-immunoprecipitated products from CD4^+^ T cells were subjected to MS analysis as described previously^33^,^34^. In agreement with previous finding, we found that DCAF1 interacted with the components of CRL4 complex including DDB1 and CUL4 in T cells (Fig. 6a and Supplementary Table 1). Of interest, the prominent DCAF1-interacting proteins in T cells belong to COP9 signalosome. The interaction of DCAF1 with CSN1 and CSN2 (two important components of COP9 signalosome) was verified by co-immunoprecipitation (co-IP) assay (Fig. 6b). Further analysis of protein–protein interaction network based on STRING database showed that COP9 signalosome subunits and CRL4 components interacted with each other (Fig. 6c). Therefore DCAF1 belongs to a mega-complex-containing multiple subunits of CRL4 complex and COP9 signalosome in T cells. COP9 signalosome is critical for T-cell function. Deletion of CSN8 (a subunit of COP9 signalosome) destabilizes the components of COP9 signalosome and leads to defective cell cycle entry in T cells^35^. It is therefore possible that DCAF1 deletion destabilized the components of COP9 signalosome. The stability of the components of COP9 signalosome, however, appeared normal in the absence of DCAF1. The protein levels of CSN1 and CSN2 were comparable between DCAF1-sufficient and DCAF1-deficient T cells (Fig. 6d). Notably, the expression of CUL4A and DDB1 and the neddylation of CUL4A^35^ appeared normal in the absence of DCAF1 (Fig. 6e). These findings therefore suggest that DCAF1 is a critical component of CRL4–COP9 mega-complex in controlling cell growth and cell cycle entry.

### DCAF1 deficiency leads to p53 protein stabilization

**Figure 7:**
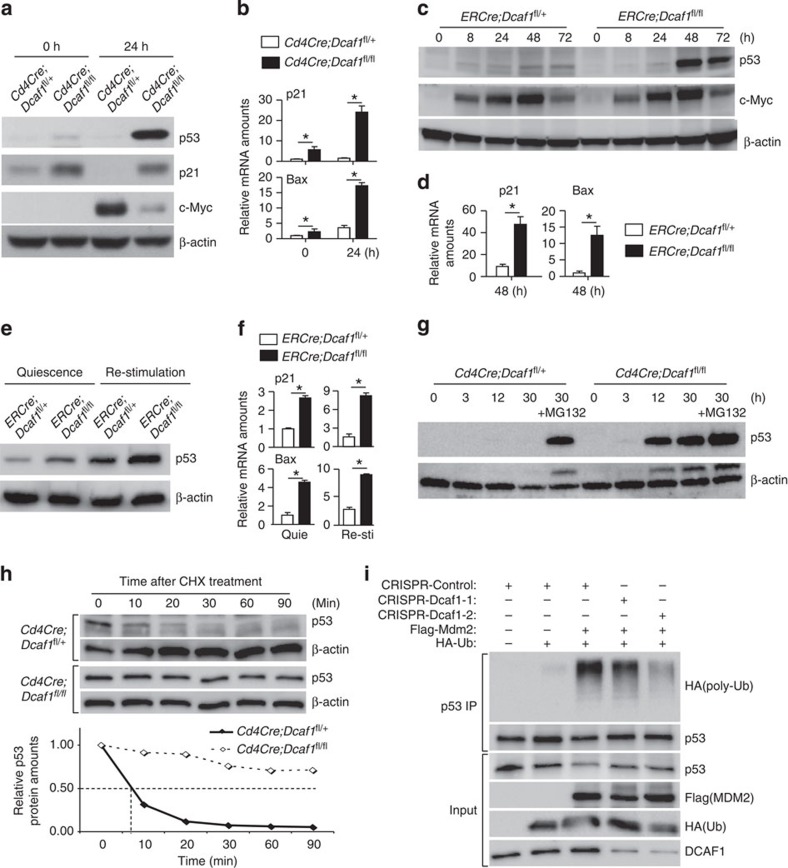
DCAF1 deficiency leads to p53 protein stabilization. (**a**,**b**) The protein expression of p53, p21 and c-Myc detected by immunoblotting (**a**) and the mRNA expression of p21 and Bax detected by quantitative reverse transcription–PCR (qRT–PCR) (**b**) in CD4^+^ naive T cells of different genotypes at indicated time points after being activated with anti-CD3 and anti-CD28. (**c**,**d**) CD4^+^ naive T cells of various genotypes were activated with anti-CD3 and anti-CD28 in the presence of 4-hydroxy-tamoxifen. At indicated time points after activation, p53 and c-Myc protein expression was determined by immunoblotting (**c**), and p21 and Bax mRNA expression was assessed by qRT–PCR (**d**). (**e**,**f**) The protein expression of p53 by immunoblotting (**e**) and mRNA expression of p21 and Bax by qRT–PCR (**f**) in the quiescent and IL-2-re-stimulated effector T cells, which were generated in the presence of 4-hydroxy-tamoxifen as described in Fig. 4e,f. (**g**) The protein expression of p53 in CD4^+^ naive T cells of different genotypes at indicated time points after being activated with anti-CD3 and anti-CD28 in the presence and absence of proteasome inhibitor MG132. (**h**) p53 protein half-life in anti-CD3- and anti-CD28-activated (for 24 h) CD4^+^ T cells of different genotypes, determined by immunoblotting for p53 in the presence of translation inhibitor cycloheximide (CHX). (**i**) Human osteosarcoma U2OS cells were transduced (GFP^+^) by lentiviruses expressing CRISPR only (Control), CRISPR-Dcaf1-1 (sgRNA) or CRISPR-Dcaf1-2 (sgRNA) and then transfected by the plasmids expressing Flag-tagged Mdm2 and HA-tagged Ubiquitin (HA-Ub) as indicated. The poly-ubiquitination of immunoprecipitated (IP) p53 was detected by immunoblotting for HA tag. In this figure, results of immunoblotting are representative of three experiments. For quantitative reverse transcription–PCR assays, means±s.d. of triplicate done in one experiment representative of three are shown (*P<0.05 by Student's *t-*test). See also Supplementary Figs 4 and 5 and Supplementary Table 2.

We further investigated whether DCAF1 deletion affected the expression of critical regulators for cell growth and cell cycle entry. Because c-Myc and p53 have been shown to be critical for the cell growth and cell cycle entry^2^,^36^,^37^,^38^, we investigated if DCAF1 deletion affected the expression of c-Myc and p53 and found that the p53 and its target p21 cell cycle inhibitor were more abundantly expressed in DCAF1-deficient than in wild-type T cells after TCR activation. In addition, TCR-activated c-Myc upregulation was reduced in DCAF1-deficient T cells (Fig. 7a and Supplementary Fig. 4a). The expression and downregulation of p27 cell cycle inhibitor was, however, unperturbed in DCAF1-deficient T cells (Supplementary Fig. 4b). In agreement with the increased p53 expression, the mRNA levels of p53 target genes including *p21* and *Bax*^39^,^40^,^41^ were elevated in DCAF1-deficient T cells (Fig. 7b), contributing to impaired cell cycle entry and increased apoptosis (Fig. 3 and Supplementary Fig. 2e). Because DCAF1 was deleted in *CD4Cre;Dcaf1*^fl/fl^ T cells long before the TCR activation, some of the DCAF1-dependent changes (Fig. 7a,b) may not be a primary effect of DCAF1 deletion. We therefore further studied how the p53 and c-Myc expression was affected when DCAF1 was acutely deleted. Using *ERCre*;*Dcaf1*^fl/fl^ T cells, we deleted DCAF1 acutely *in vitro* as described in Fig. 4a. We found that p53 protein level greatly elevated at the time of DCAF1 deletion (Fig. 7c) with increased mRNA expression of p53 target genes *p21* and *Bax* (Fig. 7d), whereas c-Myc expression did not obviously change (Fig. 7c). Therefore, increased expression of p53 was one of the primary effects of DCAF1 deletion. Using *ERCre*;*Dcaf1*^fl/fl^ T cells, we also assessed how DCAF1 deletion affected p53 expression in quiescent effector T cells and IL-2-stimulated effector T cells. p53 and its target genes including *p21* and *Bax* were expressed at higher levels in DCAF1-deficient than in DCAF1-sufficient effector T cells during quiescence and IL-2 stimulated cell cycle entry (Fig. 7e,f). Proteasome-mediated protein degradation is a primary means to control p53 expression in non-lymphoid cells^42^. We found a drastic stabilization of p53 in activated wild-type T cells treated with proteasome inhibitor MG132, although MG132 treatment did not further increase p53 amount in DCAF1-deficient cells (Fig. 7g), suggesting that DCAF1 deletion led to p53 protein stabilization. Indeed, the half-life of p53 was much longer in DCAF1-deficient than in DCAF1-sufficient T cells (Fig. 7h). Thus DCAF1 deletion leads to p53 protein stabilization.

To understand how DCAF1 regulates p53 stabilization, we analysed the DCAF1-interacting protein profile in T cells by co-IP MS analysis. DCAF1 interacted with CRL4 components and COP9 signalosome subunits in a mega-complex in CD4 T cells (Fig. 6c). Both CRL4 complex and COP9 signalosome have been reported to regulate MDM2-mediated p53 stability^43^,^44^. Further analysis showed that many DCAF1-associating proteins are known to regulate MDM2 function^45^ (Supplementary Table 2). In addition, we found that DCAF1 bound to MDM2 in activated T cells (Supplementary Fig. 4c), suggesting a potential role for DCAF1 in MDM2-promoted p53 poly-ubiquititation and degradation. To test this, we deleted DCAF1 in human osteosarcoma U2OS cells by using CRISPR-Cas9-sgRNA-mediated genome editing via lentivirus-mediated gene delivery^46^. DCAF1 deletion led to reduced expansion of U2OS cells (Supplementary Fig. 5a), increased p53 protein expression (Supplementary Fig. 5b) and substantially prolonged half-life of p53 (Supplementary Fig. 5c). Importantly, MDM2-promoted p53 poly-ubiquitination in U2OS cells was greatly reduced on DCAF1 deletion (Fig. 7i), suggesting that DCAF1 was required for MDM2-promoted poly-ubiquitination of p53 and p53 degradation. Aforementioned findings reveal a critical role of DCAF1 in mediating p53 degradation, potentially by regulating MDM2-promoted p53 poly-ubiquitination.

### p53 is required for DCAF1-controlled cell cycle entry

**Figure 8:**
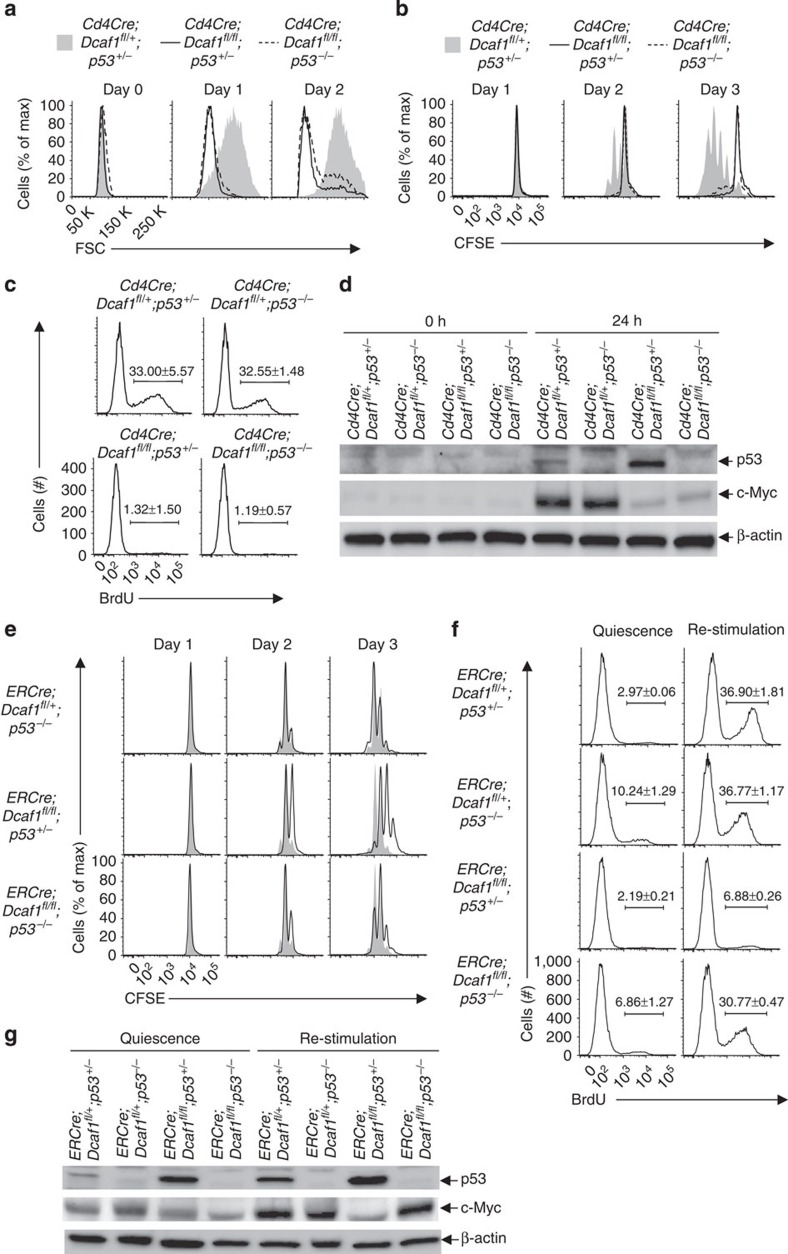
Downregulation of p53 is required for DCAF1-dependent cell cycle entry. (**a**–**d**) CD4^+^ naive T cells of different genotypes were activated by anti-CD3 and anti-CD28 at indicated time points. The cell size measured by flow-cytometry (**a**), the cell proliferation determined by CFSE dilution assay (**b**), the amounts of DNA synthesis measured by BrdU incorporation assay 24 h post activation (**c**), and p53 and c-Myc protein expression assessed by immunoblotting (**d**) were compared. (**e**) Comparison of the proliferation of CD4^+^ naive T cells of indicated genotypes (lines) to that of wild-type CD4^+^ T cells (shaded area) determined by CFSE dilution assay at indicated time points post anti-CD3 and anti-CD28 activation in the presence of 4-hydroxy-tamoxifen. (**f**,**g**) CD4^+^ naive T cells of different genotypes were activated by anti-CD3 and anti-CD28 for 5 days to generate effector T cells in the presence of 4-hydroxy-tamoxifen. Quiescent effector T cells were either re-stimulated with IL-2 for 24 h or remained unstimulated (quiescence). The amounts of DNA synthesis were determined by BrdU incorporation assay (**f**). The protein expression of p53 and c-Myc was analysed by immunoblotting (**g**). In this figure, representative flow-cytometry and immunoblotting results of three experiments are shown. For BrdU incorporation assay results (**c**,**f**), means±s.d. of three experiments are shown. See also Supplementary Fig. 6.

p53 suppresses cell growth and cell cycle entry^38^,^41^,^47^. The findings that DCAF1-deficient T cells failed to grow in size and to enter cell cycle associating with a drastic stabilization of p53 prompted us to study whether p53 is functionally important for DCAF1-controlled T-cell function. To address this question, we investigated whether and what DCAF1-dependent function(s) could be restored by deleting p53 in DCAF1-deficient cells. The *Cd4Cre*;*Dcaf1*^fl/fl^;*p53*^−/−^ mice were born with grossly normal phenotype at young age. Similar to *Cd4Cre*;*Dcaf1*^fl/fl^ mice, *Cd4Cre*;*Dcaf1*^fl/fl^;*p53*^−/−^ mice showed largely normal T-cell development in the thymus (Supplementary Fig. 6a,b) but impaired mature T-cell maintenance in the periphery (Supplementary Fig. 6b,c). On TCR stimulation, like naive *Cd4Cre*;*Dcaf1*^fl/fl^ T cells, naive *Cd4Cre*;*Dcaf1*^fl/fl^;*p53*^−/−^ T cells failed to grow in size (Fig. 8a) and to divide (Fig. 8b) with abrogated cell cycle entry (Fig. 8c). Of note, the upregulation of c-Myc, another critical factor for cell growth and cell cycle, was defective in the absence of DCAF1 regardless of p53 expression (Fig. 8d).

Nevertheless, the dispensable role of p53 in cell cycle entry of naive T cells could be due to the failed cell growth, since cell growth occurs slightly earlier than cell cycle entry in naive T cells after TCR activation (Fig. 1a–d). To specifically examine how DCAF1–p53 axis regulates cell cycle entry, we deleted DCAF1 after bypassing the growth phase in *ERCre*;*Dcaf1*^fl/fl^;p53^−/−^ T cells. Timed DCAF1 deletion in TCR-activated *ERCre*;*Dcaf1*^fl/fl^;*p53*^−/−^ T cells did not apparently affect cell growth (Supplementary Fig. 6d). We found that the cell proliferation defect of *ERCre*;*Dcaf1*^fl/fl^ T cells was substantially restored when p53 was deleted (Fig. 8e), suggesting that p53 is involved in DCAF1-dependent cell cycle regulation. Encouraged by this finding, we examined IL-2-promoted cell cycle entry of quiescent effector *ERCre*;*Dcaf1*^fl/fl^;*p53*^−/−^ T cells. In the absence of p53, DCAF1-deficient effector T cells entered cell cycle at a rate close to wild-type T cells on IL-2 stimulation (Fig. 8f). We also noticed that the c-Myc upregulation in DCAF1-deficient effector T cells during IL-2-stimulated cell cycle entry from quiescence was restored on p53 deletion (Fig. 8g). Therefore, DCAF1 controls cell cycle entry through p53. Collectively, aforementioned findings suggest that DCAF1 and p53-dependent programme controls cell cycle entry from quiescence and yet DCAF1-dependent, p53-independent programme dictates cell growth.

## Discussion

The majority of the cells in adult tissues including mature T cells are maintained in a quiescent state with a low rate of cell size growth (biomass increase due to enhanced biosynthesis) and proliferation. Disturbances of the homeostasis, such as those inflicted by injury, infection and inflammation, often trigger cells to exit quiescence and to enter cell cycle, followed by rapid proliferation to correct the disturbance and to restore homeostasis. Cell size growth is imperative for the normal function of proliferating cells to compensate for the halving of cellular constituents for each division^48^. Thus, cell growth, cell cycle entry and subsequent proliferation are fundamental processes for a cell to respond to changing microenvironment and to maintain homeostasis. In particular, proper coordination of cell growth and cell cycle entry of T cells is essential for their clonal expansion and function to mount effective immune response. Nonetheless, little is known about how cell cycle entry is controlled and even less about how cell size growth is regulated^49^,^50^. One of the difficulties to study the biochemistry and genetic requirements for cell growth versus cell cycle entry is the lack of robust experimental systems to clearly distinguish these processes because they often occur simultaneously and appear inseparable. In this study, using activated naive and effector T cells as model systems, we readily distinguished cell growth and cell cycle entry phases based on the timing, morphological, biochemical and molecular criteria. Taking advantage of such a system, we identified DCAF1 a highly upregulated factor in activated T cells and revealed a pivotal role for DCAF1 in controlling T-cell function and T-cell-mediated anti-viral and autoimmune responses.

Over-exuberant T-cell growth and proliferation are associated with and required for the development of various diseases including inflammation, autoimmunity and leukaemia. Inhibiting T-cell growth and cell cycle entry of aberrantly activated T cells are effective ways to treat these disorders. This study revealed a fundamental requirement of DCAF1 for activation-induced cell growth and cell cycle entry and showed that interfering with DCAF1 led to defective expansion of both T cells and tumour cells. Therefore, DCAF1 is central to the critical steps towards aberrant cell expansion and thus could be a useful therapeutic target for treating inflammation and cancer. In addition, because DCAF1 is a cellular protein targeted by Vpr of HIV^9^, understanding the function of DCAF1 in immune cells will shed light on how HIV evades and undermines immune function. Recent studies showed that HIV evades innate defense system in macrophages through DCAF1 (refs 10, 11). We now found that perturbation of DCAF1 led to decreased numbers of naive, effector and memory T cells (major cell types targeted by HIV) under steady state and the abrogation of T-cell response towards virus infection. These findings suggest that one way that HIV undermines T-cell function may be by interfering with DCAF1 function. It thus suggests that blocking Vpr–DCAF1 interaction may help preserve T-cell population and function against HIV.

The down-modulation of p53 by TCR signalling is critical for T-cell proliferation^38^. The molecular mechanisms underlying TCR-induced p53 downregulation are, however, unclear. This study revealed that DCAF1 was upregulated on TCR stimulation and required for p53 degradation. It therefore suggests that DCAF1 is an important factor relaying TCR signalling to modulate p53 expression for a proper T-cell proliferation and function. Because we found that DCAF1 was required to destabilize p53, a factor regulates both cell metabolism^47^ and cell cycle entry^38^,^41^, we posited that p53 was an important downstream target of DCAF1 for both cell growth and cell cycle entry. We, however, found that p53 deletion restored the cell cycle entry of DCAF1-deficient cells but had no beneficial effect on the cell growth of these cells. While the findings will not rule out a role for p53 in DCAF1-controlled cell growth, it suggests that p53-independent pathway(s) are more important. One of these pathways could involve c-Myc. c-Myc is important for both cell growth and proliferation^2^. During T-cell growth, c-Myc failed to be upregulated in the absence of DCAF1 regardless of p53 expression. Therefore, c-Myc-controlled cell growth is regulated by p53-independent programme(s) downstream of DCAF1. Nonetheless, the c-Myc expression during cell cycle entry appeared to be controlled by a DCAF1–p53-dependent programme, because p53 deletion restored c-Myc expression to a normal level in DCAF1-deficient effector T cells during cytokine IL-2-stimulated cell cycle entry from quiescence. It indicates that c-Myc expression is regulated by discrete genetic programmes during cell growth and cell cycle entry: p53 is dispensable for c-Myc expression during cell growth, but p53 is important to suppress c-Myc expression during cell cycle entry from quiescence. DCAF1–p53 signalling pathway identified in this study sheds a new light on how TCR signalling and cell cycle regulation co-opt to ensure a proper T-cell function during immune responses.

DCAF1 functions through diverse mechanisms including regulating protein poly-ubiquitination, mono-ubiquitination and phosphorylation^13^,^23^,^24^,^25^. We found that DCAF1 was required to destabilize p53 protein. One of the mechanisms underlying DCAF-controlled p53 degradation could be related to MDM2, because DCAF1 bound to MDM2 and to many proteins that are known to regulate MDM2 function. Importantly, MDM2-promoted poly-ubiquitination of p53 was impaired when DCAF1 expression was defective. In addition, because we found that DCAF1 belonged to a mega-complex-containing CRL4 complex and COP9 signalosome in T cells, it is plausible that other components of this mega-complex also regulate p53 protein stability. In fact, CUL4A (a CRL4 complex subunit), CSN2 and CSN6 (COP9 signalosome subunits) have been shown to regulate p53 degradation^43^,^44^,^51^. DCAF1 thus appears to control p53 stability through multiple mechanisms. A comprehensive understanding of the precise mechanisms underlying DCAF1-dependent control of p53 is therefore of interest warranting further studies. We found that, besides regulating p53, DCAF1 is critical for c-Myc expression independent of p53 during cell growth. The underlying mechanism, however, remains elusive at this moment. The finding that DCAF1 deletion consistently led to reduced mRNA expression of *c-Myc* during cell growth implies that DCAF1 may control c-Myc expression through factors important for transcription and/or epigenetics, for example, TET (Ten-eleven translocation methylcytosine dioxygenase) proteins^12^,^13^. Therefore, in light of the current finding that DCAF1 was critical for T-cell function by controlling cell growth and cell cycle entry via distinct molecular programmes, it is of interest to comprehensively investigate the exact mechanisms underlying the essential and diverse functions of DCAF1 during these fundamental biological processes.

## Methods

### Mice

*Dcaf1*^fl/fl^, *p53*^−*/*−^, *Rag1*^−*/*−^, *SMARTA*, *Cd4Cre*, *ERCre* and CD45.1 congenic wild-type mice were on C57BL/6 background. All mice were housed and bred under specific pathogen-free conditions in the animal facility at the University of North Carolina at Chapel Hill. Eight- to 16-week old, age- and sex-matched mice were used for animal experiments. All mouse experiments were approved by Institution Animal Care and Use Committee of the University of North Carolina.

### Flow-cytometry and cell sorting

Lymphocytes were isolated from the thymus, spleen and peripheral lymph nodes of age- and sex-matched mice of 8–16 weeks of age. Fluorescence-conjugated antibodies for CD4 (Cat#100428, 1:400), CD8 (Cat#100722, 1:400), CD45.1 (Cat#110718, 1:400), CD45.2 (Cat#109814, 1:400), CD25 (Cat#102008, 1:400), CD44 (Cat#103016, 1:400), CD62L (Cat#104412, 1:400) and CD69 (Cat#104508, 1:400) were purchased from Biolegend. Annexin V (BD Biosciences, 550474) and 7AAD (BD Biosciences, 559925) staining were used to assess apoptosis. The staining was performed as per manufacturer's protocols. For cell sorting, CD4^+^ T cells were enriched by MACS (Miltenyi Biotec, 130-049-201) and then stained with fluorescence-conjugated antibodies. Stained cells were either analysed on the LSRII station (BD Biosciences) or sorted on the Moflow cell sorter (Dako cytomation, Beckman coulter) by the flow facility of the University of North Carolina at Chapel Hill. FACS data were analysed with FlowJo software (TreeStar).

### T-cell activation and proliferation

T cells were activated with anti-CD3 (2c11) and anti-CD28 (27.51) in Bruff's medium supplemented with 10% (vol/vol) FBS and 1% (vol/vol) penicillin/streptomycin. For carboxyfluorescein succinimidyl ester (CFSE) dilution assay, T cells were labelled in 2 μM CFSE (Life Technologies, C1157) and cultured in the presence of 2 ng ml^−1^ IL-2 (BioLegend, 575406). For BrdU incorporation assay, cultured T cells were pulsed with BrdU for 1 h prior to harvest, stained with BrdU staining kit as per manufacture's protocol (BD Pharmingen, 559619) and analysed by flow-cytometry. For cells from *ERCre*;*Dcaf1*^fl/fl^ mice, 1 μM of 4-hydroxy-tamoxifen (Sigma, H7904) was added to the culture to induce the deletion of the floxed *Dcaf1* alleles at the time of T-cell activation. For p53 protein expression assay, 20 μM of MG132 (Santa Cruz, sc-201270A) was added into cell culture 4 h prior to harvest. For p53 half-life assay, 1 μg of cycloheximide (CHX) (Sigma, C7698) was added into cell culture medium at the indicated time point.

### Knockout of DCAF1 by CRISPR/Cas9 system in human U2OS cells

Construction of CRISPR-DCAF1 plasmids: DNA oligos targeting the protein-coding region of human *Dcaf1* (Dcaf1-1: 5′-ATGTACCACTACTGTAGTCA-3′; Dcaf1-2: 5′-GACTACAGTAGTGGTACATG-3′) were ligated into lentiviral vector Lenti-sgRNA-Cas9–GFP, which was modified from LentiCRISPR^52^ by replacing puromycin-resistance gene with green fluorescent protein (GFP) gene.

### CRISPR-Dcaf1 and CRISPR-control lentivirus production and enrichment

HEK293T cells were transfected with 15 μg of lentiCRISPR plasmids, 15 μg of packaging plasmids (5 μg of psPAX2, 10 μg of pMD2.G) using a calcium phosphate transfection reagent. After 48 h, viruses were harvested, filtered through 0.45-μm syringe filters and concentrated by ultracentrifugation at 26,000 r.p.m. in a Beckman SW28 ultracentrifuge rotor for 90 min at 4 °C and then resuspended with 1 ml MyCoy's Medium.

### Cell transduction and transfection

For transduction of U2OS cells with CRISPR-Dcaf1 lentivirus, U2OS cells were seeded onto a 24-well plate at a concentration of 0.75 × 10^5^ per well on day 0. Transduction was performed in fresh media supplemented with 8 μg ml^−1^ polybrene (Sigma-Aldrich, AL-118) on day 1. Transduced U2OS cells were reseeded onto a 10-cm dish on day 3 and transfected with pCDNA–Flag–MDM2 and pCMV–HA–Ubiquitin plasmids using FuGENE 6 transfection reagent on day 4. Cells were treated with 20 μM of proteasome inhibitor MG132 (sc-201270A) for 4 h before harvest on day 6.

### Bone marrow chimera

Bone marrow cells were isolated from age- and gender-matched *Cd4Cre;Dcaf1*^*fl/fl*^ mice (CD45.2^+^) and wild-type mice (CD45.1^+^CD45.2^+^). Bone marrow cells of different origins were mixed at the ratio of 1:1 and transferred retro-orbitally into sub-lethally irradiated (500 cGy) wild-type (CD45.1^+^) or *Rag1*^−*/*−^ mice. T-cell population and function analysis were performed at 8–10 weeks post cell transfer.

### T-cell adoptive transfer and LCMV infection

CD4^+^ T cells were purified from *Cd4Cre;Dcaf1*^*fl/+*^;*SMARTA* (CD45.2^+^) and *Cd4Cre;Dcaf1*^*fl/fl*^*;SMARTA* (CD45.1^+^CD45.2^+^) mice by MACS. Cells of different origins were mixed at the ratio of 1:1 and then transferred retro-orbitally into wild-type mice (CD45.1^+^). Twenty-four hour post transfer, recipient mice were injected i.p. with 2 × 10^5^ p.f.u. of LCMV (Armstrong strain). T-cell populations in the spleens were assessed by flow-cytometry 5 days after the virus infection.

### Eliciting EAE

Mice (12- to 16-week old) were immunized s.c. with 50 μg of MOG_35–55_ peptide (MEVGWYRSPFSRVVHLYRNGK, AnaSpec) and 500 μg of *Mycobacterium tuberculosis* (Difco) emulsified in incomplete Freund's adjuvant (IFA) (Difco). In addition, the animals received 200 ng of pertussis toxin (List Biological Laboratories) i.p. on days 0 and 2. The severity of EAE was monitored and graded on a clinical score of 0 to 5: 0=no clinical signs; 1=limp tail; 2=paraparesis (weakness, incomplete paralysis of one or two hind limbs); 3=paraplegia (complete paralysis of two hind limbs); 4=paraplegia with forelimb weakness or paralysis; 5=moribund or death. The statistical significance was analysed by nonparametric Mann–Whitney's *U*-test.

### Immunoblotting IP and MS

Protein extracts were resolved by AnyKD SDS–PAGE gel (Bio-Rad, 4569034) and transferred to a polyvinylidene fluoride membrane (Millipore) and analysed by immunoblotting with the following antibodies: β-actin, Santa Cruz (I-19), WB (1:2,000); Rb, BD Pharmingen (G3-245), WB (1:2,000); DCAF1, ProteinTech (11612-1-AP), WB (1:2,000), IP (1:200); p53, Leica (IMX25), WB (1:2,000), IP (1:200); p21 Santa Cruz (F-5), WB (1:500); c-Myc, Cell Signaling (D84C12), WB (1:2,000); p27, Cell Signaling (SX53G8.5), WB (1:2,000); Cyclin D3, Cell Signaling (DCS22), WB (1:1,000); Cyclin E, Cell Signaling (HE12), WB (1:1,000); MDM2 (2A10), gift from Dr Yanping Zhang, WB (1:200), IP (1:50). Images of western blot have been cropped for presentation. Full size images are presented in Supplementary Fig. 7.

To identify DCAF1-interacting proteins in T cells, CD4 T cells were purified by MACS and activated by plate bound anti-CD3 and anti-CD28 for 24 h. The cells were treated with MG132 for 4 h prior to harvest. Cells were lysed in NP40 lysis buffer (1% Nonidet P-40, 50 mM Tris (pH 7.5), 150 mM NaCl, 10% glycerol) containing protease inhibitor cocktail (Roche Molecular Biochemicals) and MG132. The crude lysates were cleared by centrifugation at 14,000 r.p.m. at 4 °C for 15 min. The supernatant was divided into two parts and incubated with agarose beads (Santa Cruz, sc-2003) that conjugated with DCAF1 antibody or rabbit IgG in cold room overnight. The immunocomplex was washed four times with NP40 lysis buffer and then three times with PBS. Associated proteins were eluted by adding 2 × Laemmli sample buffer (Bio-Rad, 1610737) and incubated at 95 °C for 5 min. The eluted proteins were resolved in SDS–PAGE gel. Protein samples were excised into 20 slices according to molecular weight and subjected to in-gel digestion with trypsin (Promega, V5113). The gel extracts were dried in a SpeedVac lypholizer and stored at −80 °C until further analysis.

The products of immunoprecipitation were then subjected to MS. MS analyses were performed using an LTQ Orbitrap Velos (Thermo Scientific, Bremen, Germany) coupled with a nanoLC-Ultra system (Eksigent, Dublin, CA). Peptides were resuspended in 30 μl HPLC buffer A (0.1% formic acid in water) and 5 μl was loaded onto an IntegraFrit column (New Objective, C18). The peptides were eluted at a flow rate of 250 nl min^−1^ with a linear gradient from 10 to 40% buffer B (0.1% formic acid in acetonitrile) for 130 min, followed by 5% buffer B for another 10 min. The MS was programmed to acquire spectra in a data-dependent and positive ion mode at a spray voltage of 2.1 kV using the XCalibur software (version 2.1, Thermo Scientific). Survey scans were performed in the Orbitrap analyser at a resolution of 60,000 over a mass range between *m/z* 300–1,800. For each cycle, the top 15 most intense ions were subjected to CID fragmentation in the LTQ Orbitrap with normalized collision energy at 35% and activation Q 0.25; dynamic exclusion was enabled. Selected ions were repeated once and then excluded from further analysis for 60 s. Unassigned ions or those with a charge of 1+ were rejected. Maximum ion accumulation times were 400 ms for each full MS scan and 200 ms for tandem MS (MS/MS) scans. One microscan was acquired for each MS and MS/MS scan.

All the raw files obtained from the LTQ Orbitrap were processed through the Andromeda and MaxQuant software suite (version 1.2.2.5) with the default settings. The MS/MS spectra were searched against UniProt mouse database (release date: 30 November 2010; 20,248 entries). A database that contains contamination proteins was also used. Peptides that matched the contaminated protein database were excluded. Trypsin was selected as enzyme and a maximum of two missed cleavage peptides was allowed. Oxidation (M) was set to be a variable modification. Peptide and MS/MS tolerance were set to be 7.5 p.p.m. and 0.5 Da, respectively. Up to two missed cleavage sites were allowed per peptide. Only peptides with a minimum length of seven amino acids were considered for identification. Peptide and protein identifications were filtered to a maximum 1% and 5% false discovery rate, respectively. The MS proteomics data have been deposited to the ProteomeXchange Consortium^53^ via the PRIDE partner repository^54^ with the data set identifier PXD003180.

### RNA preparation and real-time PCR

Total RNA was prepared from T cells using TRIzol reagent (Invitrogen, 15596-026) as per manufacturer's instructions, and was reverse-transcribed into complementary DNA with Superscript III reverse transcriptase kit (Bioline, BIO-65054). The Taqman probes of *p21* (Mm04205640_g1), *c-Myc* (Mm00487804), *Bax* (Mm00432051), *Dcaf1* (Mm01226827_g1) and *Hprt* (Mm01545399) were from Applied Biosystems and Quantitative PCR was performed using SensiFastTM Probe Lo-ROX Kit (Bioline, BIO-84020) on ABI9700 real-time PCR system.

### Statistical analysis

Data analysis was processed and presented by Prism (GraphPad, San Diego). Statistical significance was determined by Student's *t*-test unless stated otherwise. A *P* value of <0.05 was considered significant.

## Additional information

**How to cite this article:** Guo, Z. *et al.* DCAF1 controls T-cell function via p53-dependent and -independent mechanisms. *Nat. Commun.* 7:10307 doi: 10.1038/ncomms10307 (2016).

## Supplementary Material

Supplementary Information Supplementary Figures 1-7 and Supplementary Tables 1-2
