# Supplementary material for: DCAF1 controls T-cell function via p53-dependent and -independent mechanisms
Source: Nat Commun. 2016 Jan 5;7:10307. doi: 10.1038/ncomms10307 (PMC4728445; doi:10.1038/ncomms10307)
Supplement: Supplementary Information — Supplementary Figures 1-7 and Supplementary Tables 1-2 [file ncomms10307-s1.pdf]

Supplementary Fig. 1

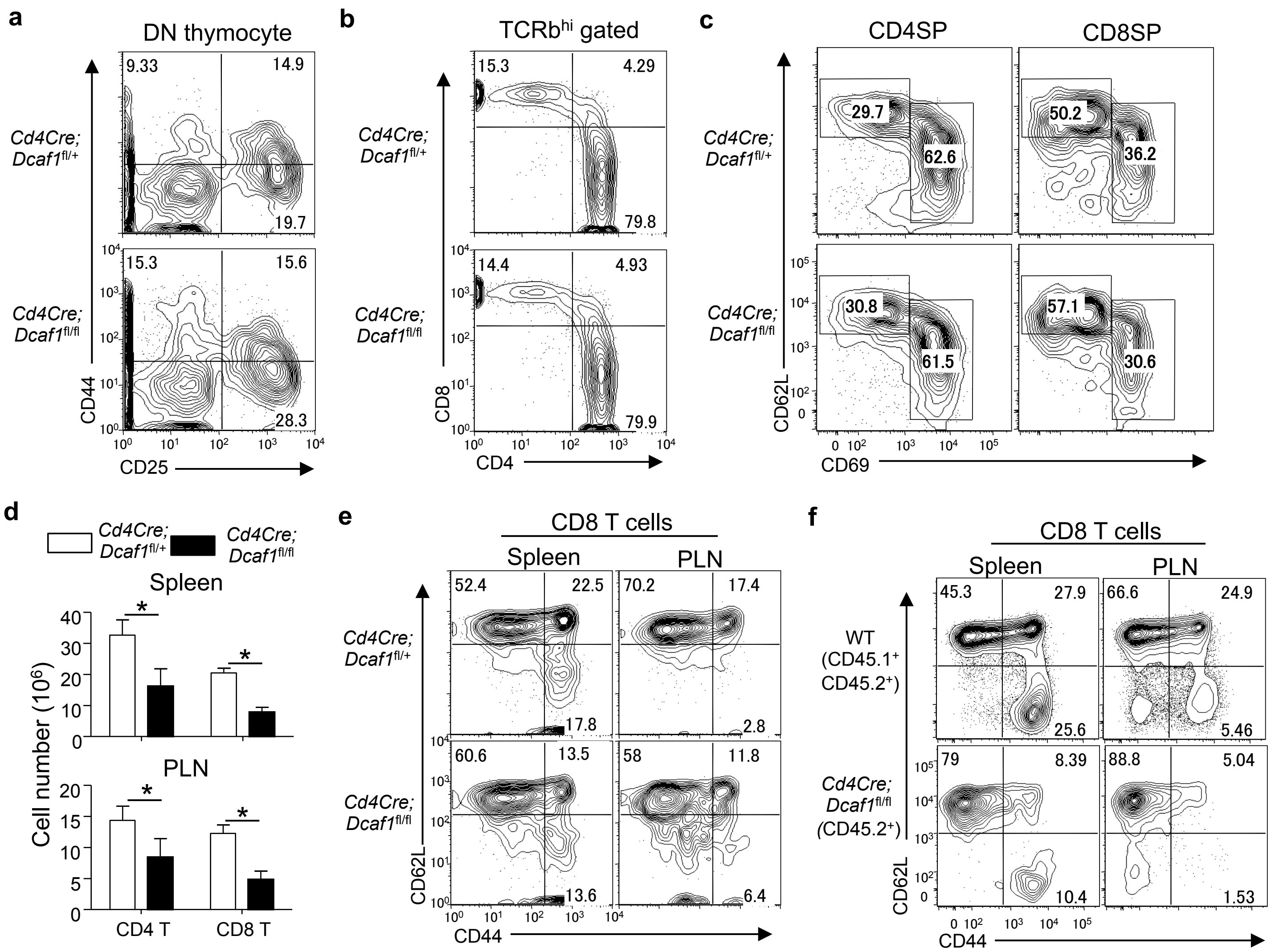

Supplementary Figure 1. The development and homeostasis of *Cd4Cre;Dcaf1*$^{fl/fl}$ T cells.

a. DN ($CD4^{-}CD8^{-}$, double negative) thymocyte development monitored by CD25 and CD44 expression assessed by flow-cytometry.

b. The expression of CD4 or CD8 in TCRb high thymocytes assessed by flow-cytometry.

c. The expression of maturation markers CD69 and CD62L on CD4 and CD8 SP (single positive) thyomcytes assessed by flow-cyometry.

d. The numbers of CD4 and CD8 T cells in the spleens and periphery lymph nodes (PLN) (right) of *Cd4Cre;Dcaf1*$^{fl/+}$ and *Cd4Cre;Dcaf1*$^{fl/fl}$ mice are counted and compared. Means ± SD of four sets of mice are shown (* P<0.05 by student's *t* test).

e. The distribution of naïve and effector CD8 T cells assessed by CD44 and CD62L staining and flow-cytometry.

f. The distribution of naïve and effector CD8 T cells of different origins in the mixed bone marrow chimeras generated as described in **Fig. 2d-2e**, analyzed by CD44 and CD62L staining and flow-cytometry analysis.

Representative results of at least three experiments are shown for **a-c** and **e-f**.

Related to Fig. 2

Supplementary Fig. 2

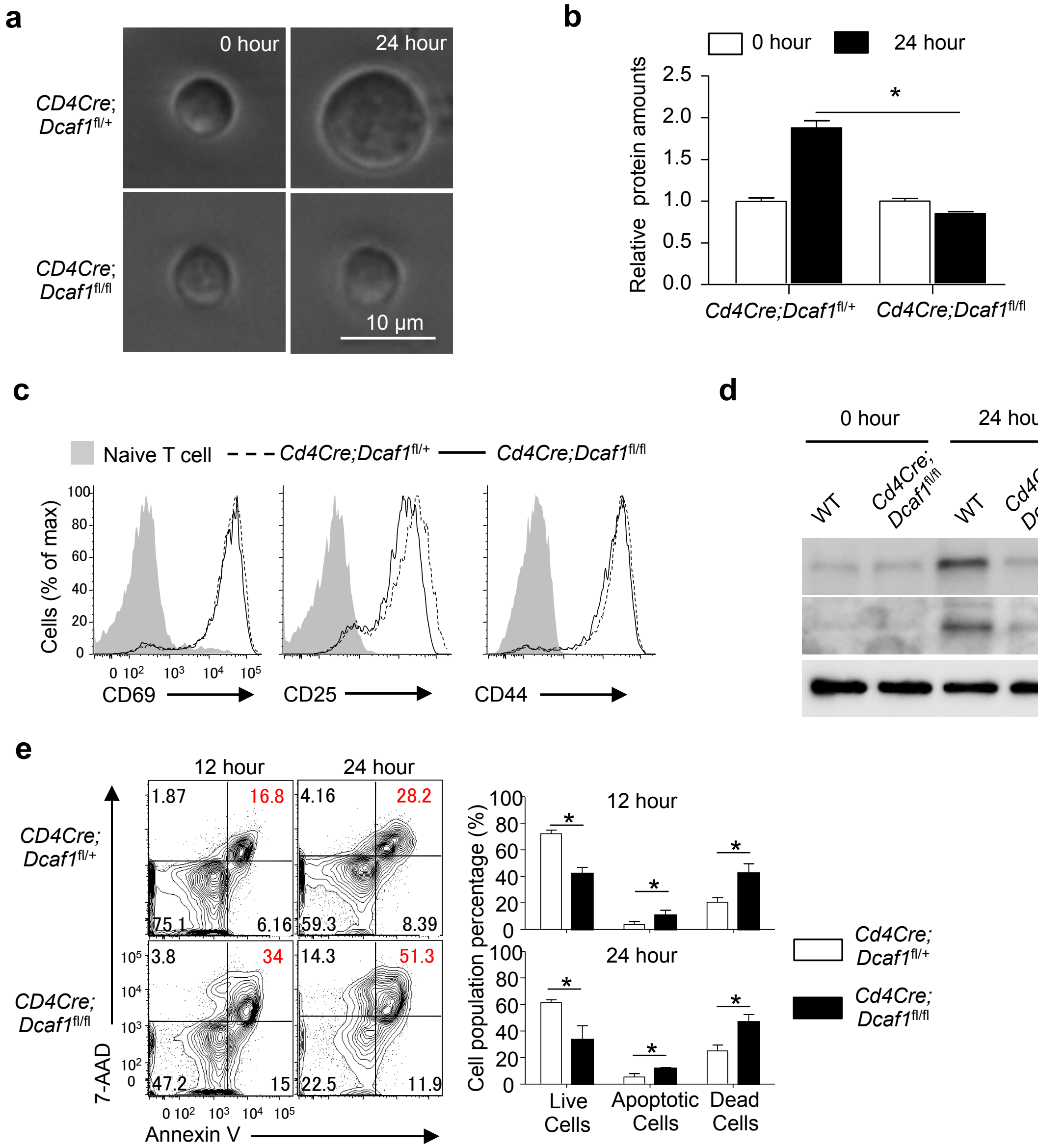

Supplementary Figure 2. DCAF1 is required for TCR-activated cell growth and cell cycle entry of naïve T cells.

a. Cell sizes analyzed by microscopy at indicated time points after naïve CD4$^+$ T cells were activated by anti-CD3 and anti-CD28.

b. Relative amounts of protein extracted from 1x10^6 CD4$^+$ T cells before and after 24 hours of TCR activation, measured by BCA assay. Means ± SD of three experiments are shown (* P<0.05 by student's *t* test).

c. The expression of activation markers including CD69, CD25 and CD44 24 hours after TCR activation, measured by flow-cytometry.

d. The protein expression of Cyclin D3 and Cyclin E was determined in CD4$^+$ T cells of different genotypes before (0 hr) and 24 hours after TCR activation.

e. The apoptosis of CD4$^+$ T cells 12 and 24 hours after anti-CD3-CD28 activation, measured by Annexin-V and 7-AAD staining and flow-cytometry. The bar graphs show the means ± SD of data from four experiments (* P<0.05 by student's *t* test).

All the results of microscopy and flow-cytometry are representative of at least three experiments.

Related to Fig. 3

Supplementary Fig. 3

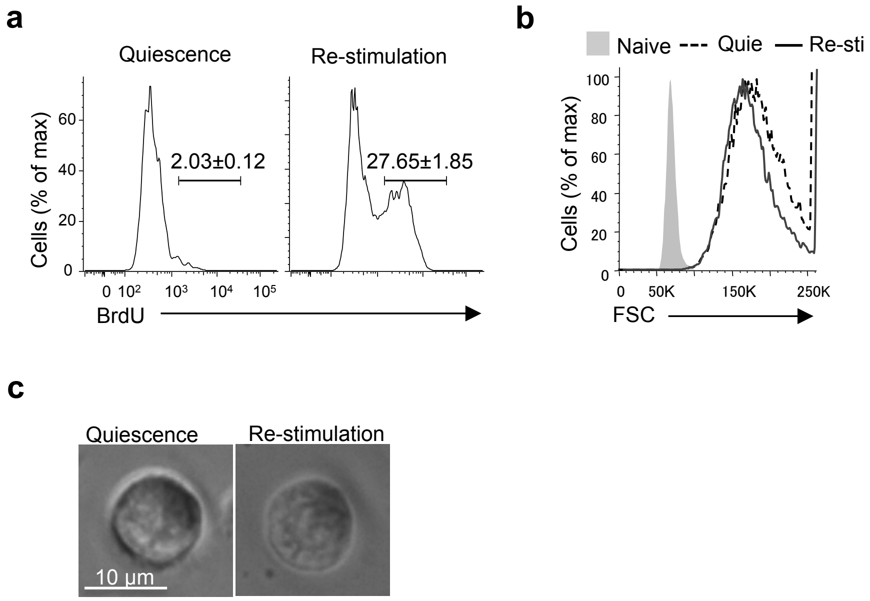

Supplementary Figure 3. Cytokine induced cell cycle entry of effector T cells.

Five days after anti-CD3 and anti-CD28 stimulation, naïve $CD4^+$ T cells became effector T cells, which were either re-stimulated with IL-2 (Re-stimulation) or remain un-stimulated (Quiescence). The amount of DNA synthesis of the effector T cells was determined by BrdU incorporation assay (**a**). The cell size was assessed by flow cytometry (**b**) and microscopic analysis (**c**). Representative results of at least three experiments and means ± SD of three experiments are shown.

Related to Fig. 4

Supplementary Fig. 4

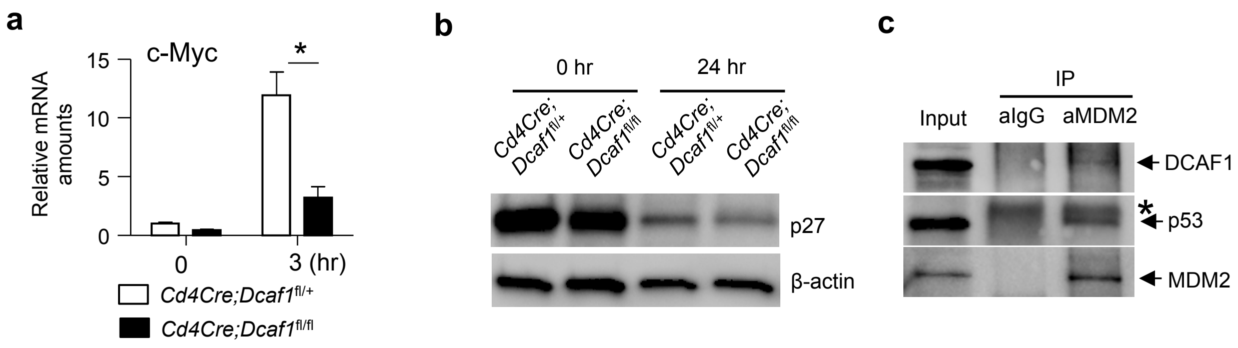

Supplementary Figure 4. DCAF1 is required c-Myc expression and binds to MDM2.

a. The mRNA expression of c-Myc in Dcaf1-sufficient (open bars) and Dcaf1-deficient (solid bards) T cells after anti-CD3 and anti-CD28 activation assessed by qRT-PCR assays. Mean ± SD of triplicates done in one experiment of at least three are shown (* $P<0.05$ by student's *t* test).
b. The protein expression of p27 in $CD4^+$ T cells of different genotypes at indicated time points after being activated with anti-CD3 and anti-CD28, assessed by immuno-blotting. Results are representative of at least three experiments.
c. The interaction between Mdm2, p53 and DCAF1 in activated wild type $CD4^+$ T cells was monitored by co-immunoprecipitation assays. Results are representative of three independent experiments. * Heavy chain of IgG.

Related to Fig. 7

Supplementary Fig. 5

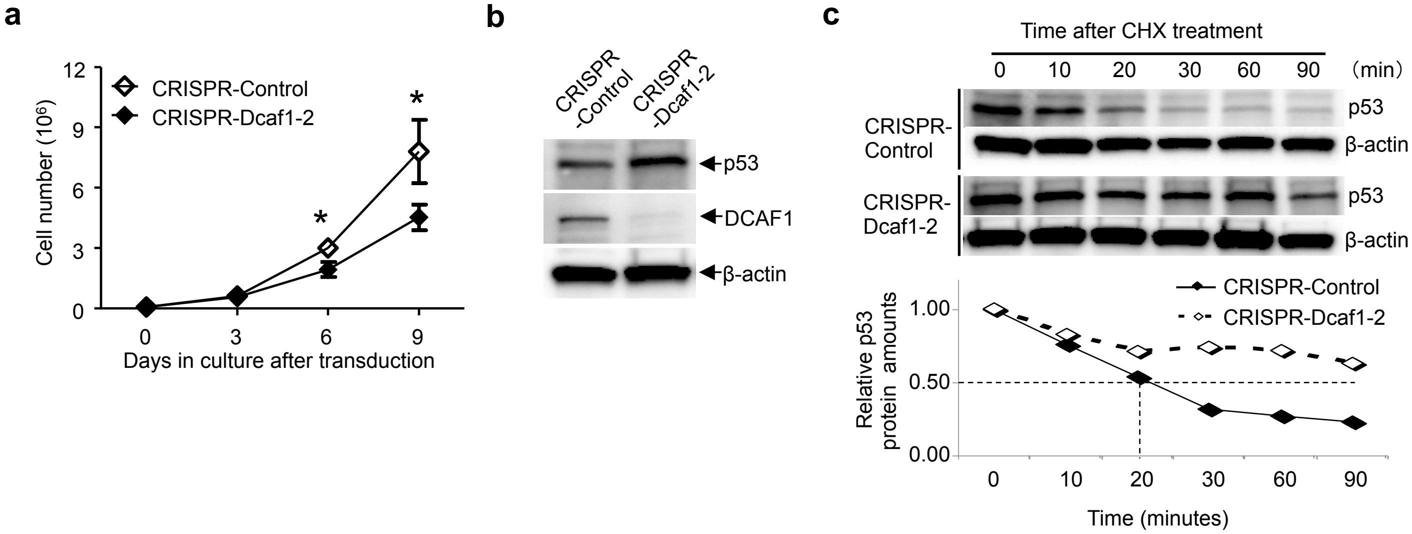

Supplementary Figure 5. DCAF1 deletion in U2OS human tumor cells leads to reduced cell expansion and p53 stabilization.

a. The numbers of recovered human U2OS cells that were transduced (GFP^+) by lentiviruses expressing CRISPR-Dcaf1-2 (sgRNA) or CRISPR-control at indicated times in the culture. Mean ± SD of triplicate in one experiment of three are shown (* $P<0.05$ by student's *t* test).

b. p53 protein expression in human U2OS cells transduced by lentiviruses expressing CRISPR-Dcaf1-2(sgRNA) or CRISPR-control detected by immuno-blotting. DCAF1 deletion deficiency was assessed by immuno-blotting. Results are representative of at least three experiments.

c. p53 protein half-life in human U2OS cells transduced by lentivirus expressing CRISPR-Dcaf1-2(sgRNA) or CRISPR-control, determined by immuno-blotting for p53 in the presence of translation inhibitor cycloheximide (CHX). Results are representative of two experiments.

Related to Fig. 7

Supplementary Fig. 6

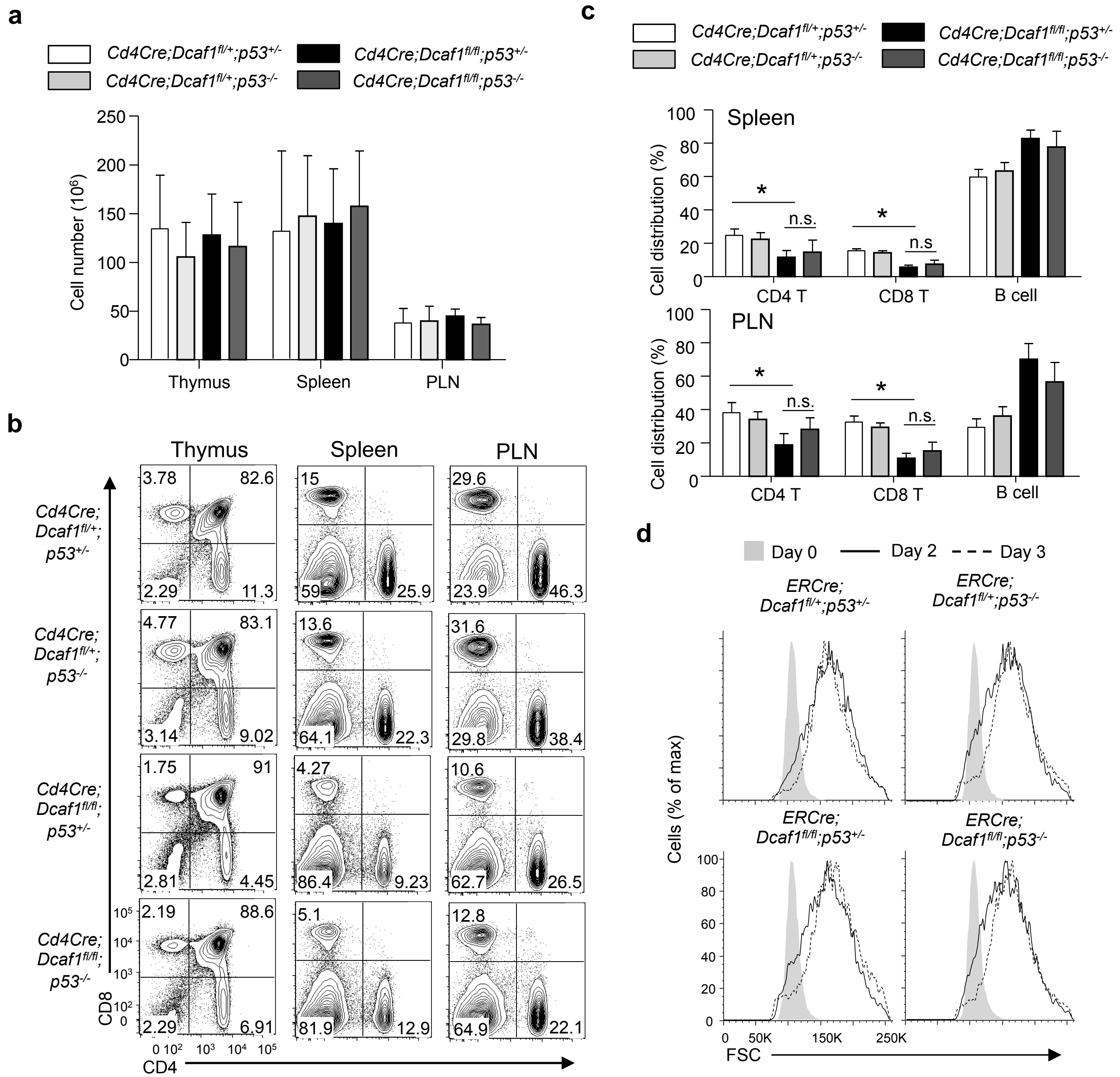

Supplementary Figure 6. T cell development and activation in the absence of DCAF1 and p53.

a. Total cell number of thymus, spleen, and periphery lymph node in the mice of different genotypes. Means ± SD of four sets of mice are shown.

b. The expression of $CD4^+$ and $CD8^+$ on the T cells in the thymi, spleens and periphery lymph nodes from mice of different genotypes, assessed by flow-cytometry. Representative results of four independent experiments are shown.

c. The percentages of $CD4^+$ T cells, $CD8^+$ T cells and B cells in the spleens and peripheral lymph nodes from mice of different genotypes, assessed by flow-cytometry. Means ± SD of four sets of mice are shown (* P<0.05 by student's *t* test; n.s. not significant, P>0.05 by student's *t* test).

d. The comparison of the sizes of the cells of different genotypes at various time points after being activated with anti-CD3 and anti-CD28 in the presence of 4-hydroxy-tamoxifen. Results are representative of three experiments.

Related to Fig. 8

Supplementary Fig. 7

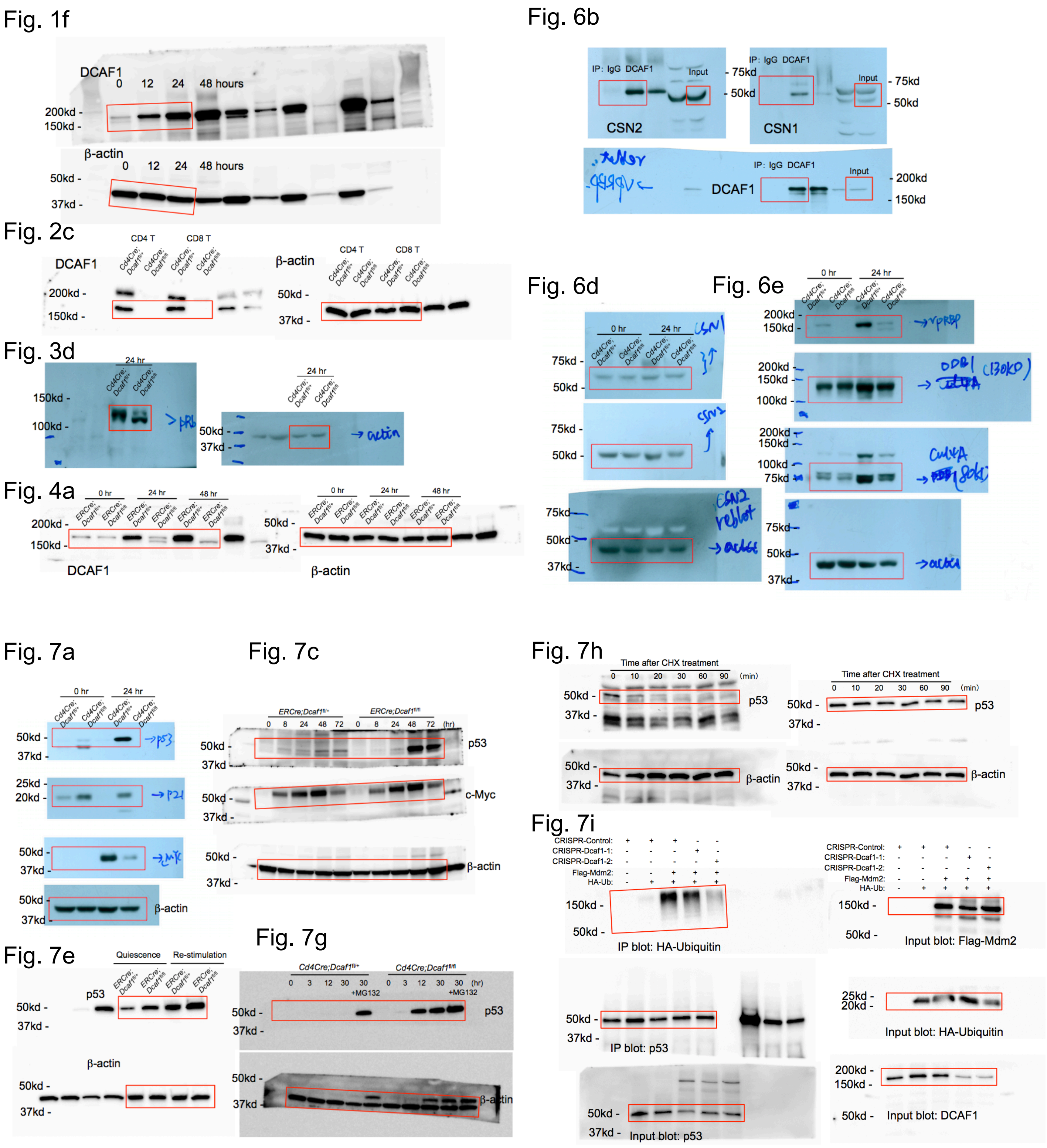

Supplementary Figure 7. Full immuno-blots with indicated areas of selection.

Supplementary Fig. 7 (continued)

Fig. 8d

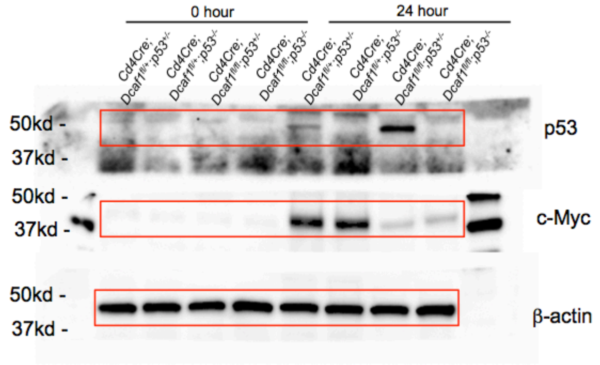

Fig. 8g

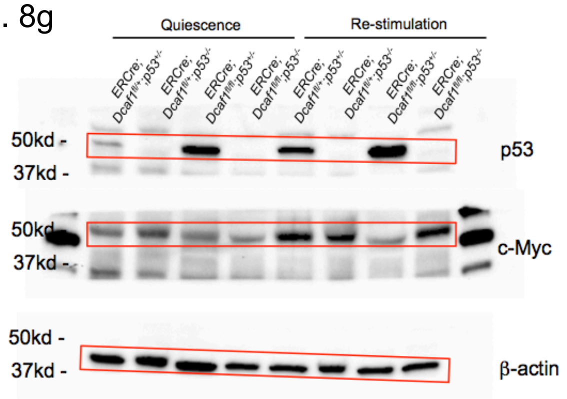

Supplementary Fig. 2d

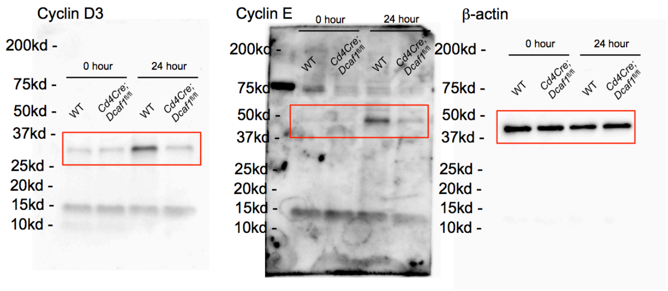

Supplementary Fig. 4c

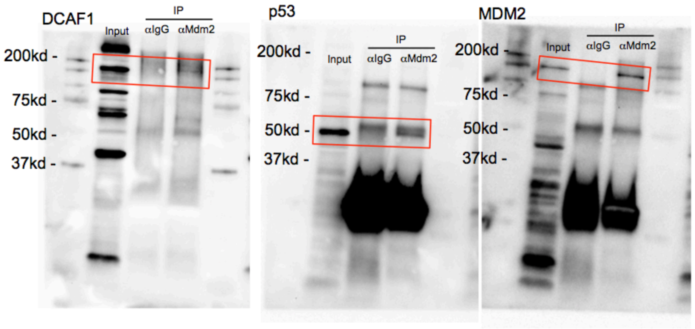

Supplementary Fig. 4b

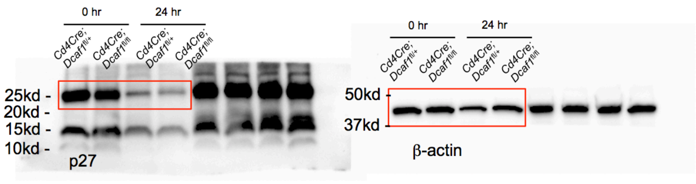

Supplementary Fig. 5c

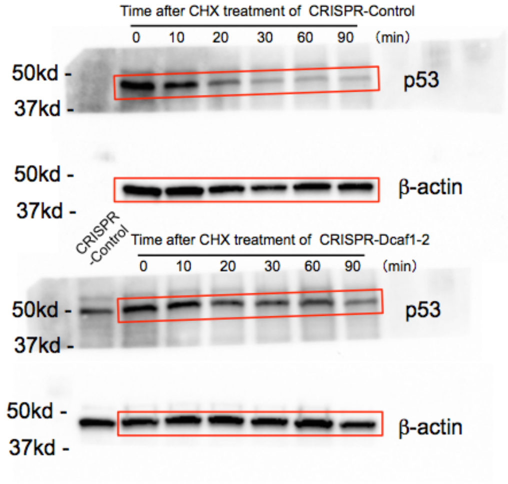

Supplementary Fig. 5b

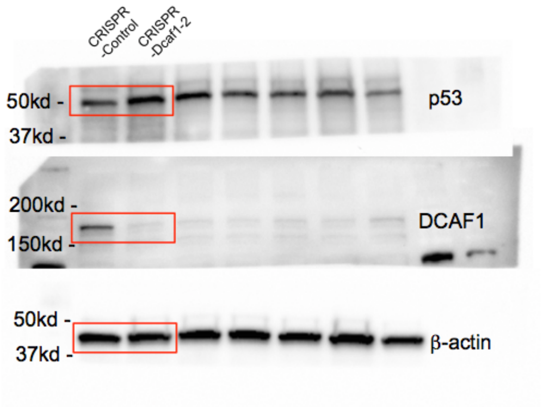

Supplementary Figure 7. Full immuno-blots with indicated areas of selection.

Supplementary table 1.

Protein Name	Peptide sequence	Protein Name	Peptide sequence
CSN1	AESTPEIAEQR	CSN7A	ELAESDFASTFR
	CAAGLAELAAR		HLSVVTLAAK
	EGSQGELTPANSQSR		QQIESEVANLK
	GHDDLGDHYLDCGDLSNALK		VTGQNQEQFLLLAK
	LFLELEPQVR		VTTAAAAAATSQDPEQHLTELR
CSN2	QLHQSCQTDDGEDDLKK	CSN7B	AGEQKPSSNLLEQFILLAK
	SGINPFDSQEAKPYK		DINNIVK
	SINSILDYISTSK		ESLPELSVAQQNK
	VLELEGEKGEWGFK		HLTIVSLASR
	WTNQLNSLNQAVVSK		TQQQVEAEVSNIKK
CSN3	ASALEQFVNSVR	CSN8	FIPLSEPAPVPPIPNEQQLAR
	CIELDER		GVLEQGWQADSTTR
	VQLSGPQEAEK		KPASGTLDVSLNR
	YATDTFAGLCHQLTNALVER		LTDYVAFLEN
	YNNPAMLHNIDQEMLK		SANSELGGIWSVGQR
CSN4	EALPTWDK	DDB1	QGQGQLVTCSGAFK
	HALHCTILASAGQQR		QSGESIDIITR
	NAAQVLVGIPLETGQK		VTLGTQPTVLR
	QLLTDFCTHLPNLPDSTAK		VVEELTR
	VISFEEQVASIR		YLAIAPPIIK
CSN5	GYKPPDEGPSEYQTIPLNK	CUL4A	EATDEELER
	ISALALLK		ESFETFINK
	LEQSEAQLGR		LQWQTTLGHAVLK
	TTIEAIHGLMSQVIK		TFGTTIVINPEK
	VNLGAFR		VITYLDHSTQKPLIACVEK
CSN6	ASEAGEVPFNHEILR	CUL4B	DKENPNQYNYIA
	ELEFLGWYTTGGPPDPSDIHVHK		EATDEELEK
	FNVLYDR		EAVEAIQNSTSIK
	IIIDKEYYYTK		ETVEEQASTTER
	QVCEIIESPLFLK		LITYLDQTTQK

Peptide sequences of DCAF1 interacting COP9 and CRL4 complex components detected by IP-MS. For each protein, 5 peptides that show high intensity are listed. Results were from three experiments.

Related to Fig. 6

Supplementary table 2.

Identified Proteins known to interact with Mdm2	Unique Peptides	Sequence coverage (%)
CSN6	13	61.6
CUL4A	23	54.8
CUL4B	46	63
DDB1	65	57.4
Elavl1(HuR)	17	62.9
GSK-3β	4	18.3
HDAC-1	13	51
HNRNPK	26	67.6
Psmd10(Gankyrin)	5	24.2
RPL11	9	44.9
RPL23	9	69.3
RPL26	11	47.6
RPL5	16	55.6
RPS14	9	41.1
RPS15	7	64.8
RPS25	6	38.4
RPS27	3	41.7
RPS7	14	67.5
TBP	4	29.3
TSG101	5	17.4

DCAF1 interacting proteins that were identified by IP-MS and are known to regulate Mdm2 function. Table summary of unique peptides and sequence coverage of DCAF1-interacting proteins identified by IP-MS. These proteins have been reported to bind to MDM2 and regulate MDM2 function. Results were generated from three experiments.

Related to Fig. 7
